# Loss of PINK1 Impairs Stress-Induced Autophagy and Cell Survival

**DOI:** 10.1371/journal.pone.0095288

**Published:** 2014-04-21

**Authors:** Dajana Parganlija, Michael Klinkenberg, Jorge Domínguez-Bautista, Miriam Hetzel, Suzana Gispert, Marthe A. Chimi, Stefan Dröse, Sören Mai, Ulrich Brandt, Georg Auburger, Marina Jendrach

**Affiliations:** 1 Experimental Neurology, Dept. of Neurology, Goethe University Medical School, Frankfurt/Main, Germany; 2 Molecular Bioenergetics Group, Goethe University Medical School, Frankfurt/Main, Germany; 3 Kinematic Cell Research Group, Institute for Cell Biology and Neuroscience, Center of Excellence Frankfurt: Macromolecular Complexes, Goethe University, Frankfurt/Main, Germany; 4 Clinic of Anesthesiology, Intensive-Care Medicine and Pain Therapy, Goethe-University Hospital, Frankfurt am Main, Germany; Istituto Nazionale per le Malattie Infettive, Italy

## Abstract

The mitochondrial kinase PINK1 and the ubiquitin ligase Parkin are participating in quality control after CCCP- or ROS-induced mitochondrial damage, and their dysfunction is associated with the development and progression of Parkinson's disease. Furthermore, PINK1 expression is also induced by starvation indicating an additional role for PINK1 in stress response. Therefore, the effects of PINK1 deficiency on the autophago-lysosomal pathway during stress were investigated. Under trophic deprivation SH-SY5Y cells with stable PINK1 knockdown showed downregulation of key autophagic genes, including Beclin, LC3 and LAMP-2. In good agreement, protein levels of LC3-II and LAMP-2 but not of LAMP-1 were reduced in different cell model systems with PINK1 knockdown or knockout after addition of different stressors. This downregulation of autophagic factors caused increased apoptosis, which could be rescued by overexpression of LC3 or PINK1. Taken together, the PINK1-mediated reduction of autophagic key factors during stress resulted in increased cell death, thus defining an additional pathway that could contribute to the progression of Parkinson's disease in patients with PINK1 mutations.

## Introduction

Parkinson's disease (PD) is the second most common neurodegenerative disorder after Alzheimer's disease and both are age-progressive disorders. PD patients are characterized by a typical impairment of their movements and resting tremor caused predominantly by degeneration of the dopaminergic neurons, which project from the substantia nigra in the midbrain to the striatum. Another hallmark of PD is the occurrence of multi-protein aggregates in the affected neurons, the so-called Lewy bodies that contain the PD-associated protein alpha-synuclein and many additional proteins.

Most PD cases occur sporadically, with aging being the main risk factor for PD. However, an increasing number of gene mutations are being associated with PD. At the moment 18 gene loci are described as PD-associated, amongst others mutations in the genes PARKIN and PTEN induced putative kinase 1 (PINK1) result in autosomal recessive PD variants PARK2 and PARK6 [1]. Different causes are hypothesized to initiate or contribute to neuronal cell death in patients with PARK6 mutations: oxidative stress [2], impaired bioenergetics [3], [4], dysregulation of neuronal Ca^2+^ [5], [6], reduced mitochondrial dynamics [7] and dysfunctional degradation of damaged mitochondria and/or protein aggregates [8], [9].

All these hypotheses implicate a progressive mitochondrial dysfunction as common denominator, which could be enforced by stress and/or impaired quality control, finally resulting in cell death. Dopaminergic neurons seem to react especially sensitively to mitochondrial dysfunction, perhaps due to their low glycolytic capacity [10], but also non-neuronal cells as e.g. skin fibroblasts from PARK6 patients demonstrate impaired mitochondrial function [2], [11].

Damaged mitochondria can be either repaired by mitochondrial dynamics (fusion and fission) or degraded by mitophagy/macroautophagy. The selection of the appropriate pathway depends on the extent of mitochondrial damage. A strong reduction of mitochondrial membrane potential induces the PINK1-regulated translocation of Parkin to these mitochondria, tagging them for degradation [12]–[15]. The actual autophagic process is mediated and regulated by the proteins of the ATG family. It starts with the engulfment of a damaged mitochondrion or protein aggregate with an expanding membrane that is characterized by the presence of the autophagosomal marker protein LC3-II (ATG8). The mature autophagosome fuses subsequently with endosomes and lysosomes to form an autolysosome. In the autolysosome the content is degraded by lysosomal hydrolases, thus providing the cell with amino acids. Lysosomes and autolysosomes are characterized by the presence of the proteins LAMP-1 and LAMP-2.

The recently emerging key roles of PINK1 and Parkin in mitophagy imply that dysfunctional mitochondrial degradation is contributing to the progression of the autosomal recessive PD variants PARK2 and PARK6, which might be enhanced by additional stressors as e.g. aging. In accordance with this hypothesis the loss of functional PINK1 or Parkin results in impaired mitophagy after stress and an accumulation of damaged mitochondria [12]–[14]. In addition to targeted mitophagy, PINK1 and Parkin are also involved in the stress response to starvation. Recent data indicate that shortage of amino acids activates general autophagy in parallel with an induction of PINK1 transcription [16], [17], indicating a role for PINK1 also in the trophic stress response. Thus, we investigated how PINK1 deficiency affects cellular and mitochondrial fitness in response to different stressors. Analyzing different cell model systems with reduced PINK1 levels, we found that reduced PINK1 expression in the presence of additional stress compromises the autophago-lysosomal pathway and results in increased cell death.

## Results

### PINK1 expression in SH-SY5Y cells is induced by activation of autophagy

**Figure 1:**
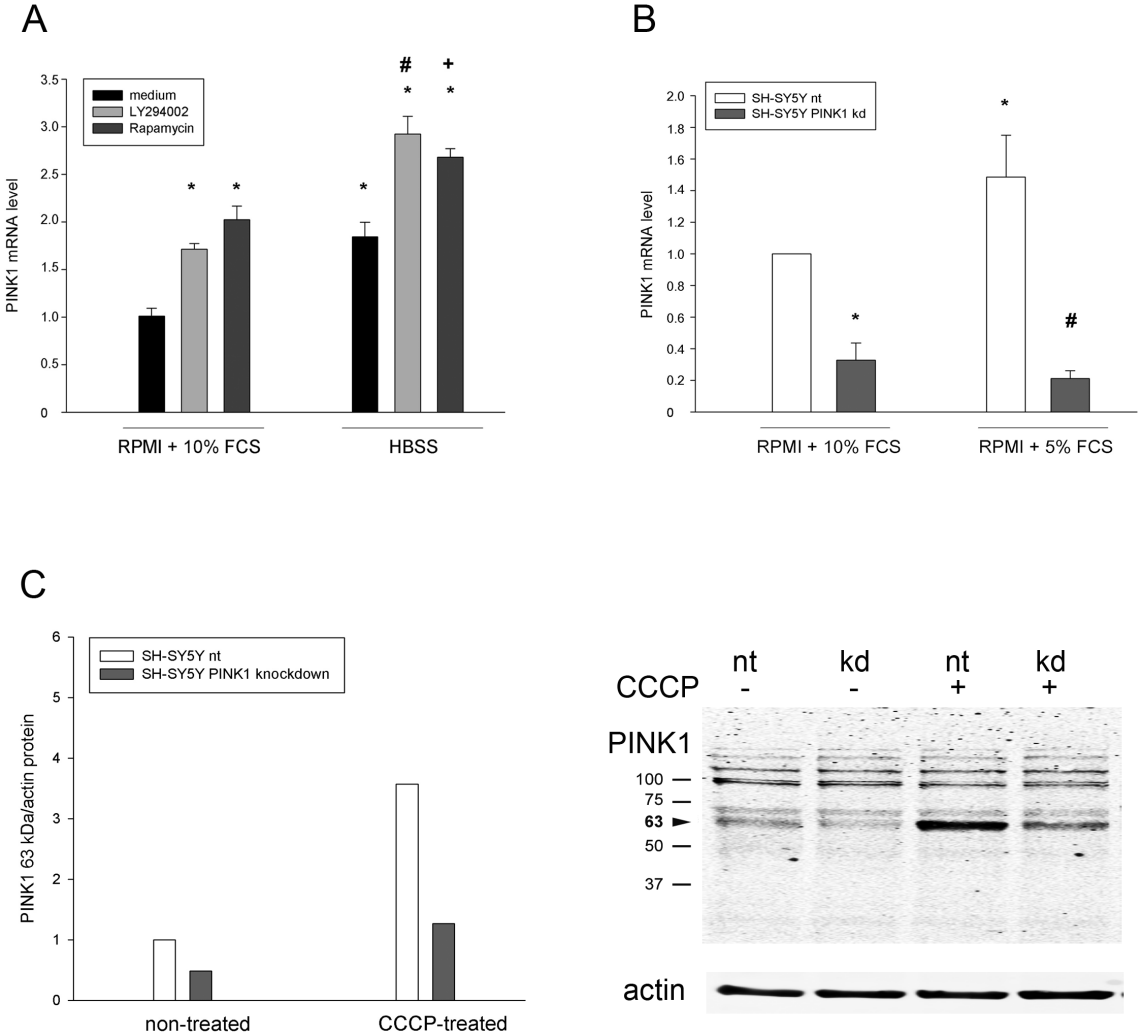
Stable PINK1 knockdown in SH-SY5Y cells. A) SH-SY5Y neuroblastoma cells were either cultivated in RPMI+10% FCS or starved in HBSS with or without addition of LY294002 or rapamycin. After 16 h PINK1 mRNA expression was determined by RT-qPCR and the PINK1 mRNA content of cells kept in RPMI+10% FCS was set as 1. Induction of autophagy by starvation or rapamycin resulted in an induction of PINK1 expression, comparable to the positive control LY294002; n = 4; * expression changes compared to the PINK1 expression in RPMI+10% FCS: LY294002: p<0.01; rapamycin: p<1×10^−4^; HBSS: p<0.005; HBSS+LY294002: p<5×10^−6^; HBSS+ rapamycin: p<0.0005; # expression changes compared to the PINK1 expression in RPMI+10% FCS+LY294002: p<0.05; **+** expression changes compared to the PINK1 expression in RPMI+10% FCS+rapamycin: p<0.0005. B) SH-SY5Y cells were either stably transduced with a control (nt) shRNA or a shRNA directed against PINK1 and cultivated in RPMI medium containing 5% or 10% FCS. Their PINK1 mRNA content was determined by RT-qPCR and PINK1 mRNA content of nt cells kept in medium with 10% FCS was set as 1. Serum reduction increased PINK1 mRNA in nt cells in accordance with the data shown in Fig. 1A, while stable PINK1 knockdown (kd) reduced PINK1 content under both conditions; n = 3; * expression changes compared to the PINK1 expression in RPMI+10% FCS: PINK1 kd 10% FCS p<0.0005; PINK1 induction by 5% FCS: p<0.05; # expression changes compared to the PINK1 expression in RPMI+5% FCS: PINK1 kd 5% FCS: p<0.005. C) SH-SY5Y cells without (nt) or with stable PINK1 knockdown (kd) were kept in medium with 5% FCS and either untreated or treated for 2 h with CCCP to stabilize PINK1. Afterwards the PINK1 63 kDa protein (arrowhead) and actin protein levels were determined by western blotting (see representative gel on the right). The quantification revealed a reduction of PINK1 protein under both conditions in PINK1 kd cells.

First, the PINK1 mRNA expression in an established PD model system, SH-SY5Y neuroblastoma cells, was analyzed in reaction to two known autophagy inducers. 16 h starvation (HBSS: amino acid free medium) or addition of rapamycin to full growth medium (RPMI+10% FCS) resulted in an about two-fold induction of PINK1 expression, comparable to the positive control LY294002, a PI3K inhibitor and known inducer of PINK1 expression [17] (Fig. 1A). Combination of starvation and rapamycin had an additive effect on PINK1 mRNA transcription, indicating a dose-dependence of PINK1 induction in respect to trophic stress and autophagy activation.

In order to identify downstream targets of PINK1 involved in the stress response to starvation, we generated transduced SH-SY5Y cells with stable PINK1 knockdown and the respective control cells as described in Material and Methods. Cells were cultivated for 3 weeks in parallel in RPMI medium supplemented with either 10% or 5% FCS, where the lowered serum content represented nutrient reduction. In accordance with the results presented above the PINK1 mRNA expression of control non-targeted (nt) shRNA SH-SY5Y cells was increased significantly in cells kept in 5% FCS (Fig. 1B). The PINK1 content of cells with stable PINK1 knockdown (kd) was significantly reduced compared to the respective nt cells in both conditions. For validation of the PINK1 knockdown, nt and PINK1 kd cells were cultivated in medium with 5% serum and either non treated or treated with 10 nM CCCP for 2 h. Western blotting showed that in both conditions PINK1 expression was reduced in PINK1 kd cells. This experiment has been performed two times and one representative result is shown in Fig. 1C.

### SH-SY5Y cells with stable PINK1 knockdown exhibit changes in autophagy gene expression

In order to determine downstream effects of reduced PINK1 expression in the stress response to nutrient deprivation, a careful comparison of diverse candidate gene transcripts in nt and PINK1 kd cells cultivated in different media was performed (Table 1). PINK1 kd cells cultivated in growth medium with 10% serum revealed no significant changes in their gene expression compared to nt cells cultivated under the same conditions. In accordance with a role of PINK1 in the stress response to trophic signaling, many key factors involved in mitochondrial dynamics and the autophago-lysosomal pathway showed significant downregulation when PINK1 kd cells were cultivated in growth medium with 5% FCS and were compared to nt cells cultivated under the same conditions. These genes included Mfn2 [18], Beclin [19], and LC3 [20], which interestingly have been all described as PINK1 interaction partners on the protein level. However, their genetic regulation by PINK1 presents a new discovery. Another PINK1-regulated gene is LAMP-2, which is also involved in the autophago-lysosomal pathway and which had not been linked to PINK1 so far. LAMP-2 showed a significantly reduced expression in PINK1 kd cells cultivated with 5% FCS, while interestingly LAMP-1 appeared not to be altered. Furthermore, several non-lysosomal, non-mitochondrial genes and two common house-keeping genes (actin and GAPDH) exhibited no PINK1-dependent changes when cultivated with 5% FCS (Table S1 in File S4).

**Table 1:** Reduced expression of autophagy genes after stable knockdown of PINK1.

Gene	PINK1 kd cells in RPMI+10% FCS Fold-change *p*-value	PINK1 kd cells in RPMI+5% FCS Fold-change *p*-value	PINK1 kd cells in HBSS for 24 h Fold-change *p*-value
*Autophagy*			
ATG5	0.991 ns	0.961 ns	0.868 0.0011
ATG6/Beclin	0.763 ns	0.579 0.0011	0.659 0.0044
ATG7	0.836 ns	0.405 0.0007	0.912 ns
ATG8E/LC3A	1.472 ns	0.32 0.0003	0.941 ns
P62/SQSTM1	1.202 ns	0.381 0.0026	1.256 ns
PARK2	1.179 ns	0.522 0.0039	0.621 0.0172
LAMP-1	1.117 ns	0.858 ns	0.895 ns
LAMP-2	0.992 ns	0.568 0.0175	0.709 0.0238
*Mitochondrial dynamics*			
Mfn1	0.948 ns	0.645 0.0018	0.771 0.0045
Mfn2	1.607 ns	0.197 0.0003	0.831 0.0067
Opa1	0.998 ns	1.077 ns	0.769 0.017
Drp1	0.882 ns	0.619 0.0107	0.752 0.0024
Fis1	0.773 ns	0.483 0.0077	0.663 6.3×10^−5^
March5	0.878 ns	0.799 0.0056	0.737 0.0006

Key genes of autophagy and mitochondrial dynamics were analyzed by RT-qPCR in control (nt) and PINK1 knockdown SH-SY5Y cells cultivated for at least 3 weeks with 10% or 5% FCS or for 24 h with HBSS. The relative gene expression in nt cells was set as 1. Several genes show only during trophic deprivation a significantly decreased expression in PINK1 knockdown cells; n = 3–4, Opa1 and LAMP-1 5% FCS: n = 7; ns: not significant.

In addition, the gene expression after short-term trophic stress was analyzed. PINK1 kd and nt cells were starved for 24 h in HBSS medium that contains 1 g/l glucose but no amino acids. Again, several genes involved in autophagy and mitochondrial dynamics exhibited a significantly reduced expression after PINK1 knockdown. In view of recent data indicating that the impairment of autophagy and lysosomes could participate in the development and progression of PD, we investigated autophagy activation and the role of LAMP-2 in the PINK1-mediated stress response.

### Impaired LC3-II expression in different cell model systems with reduced PINK1 levels

**Figure 2:**
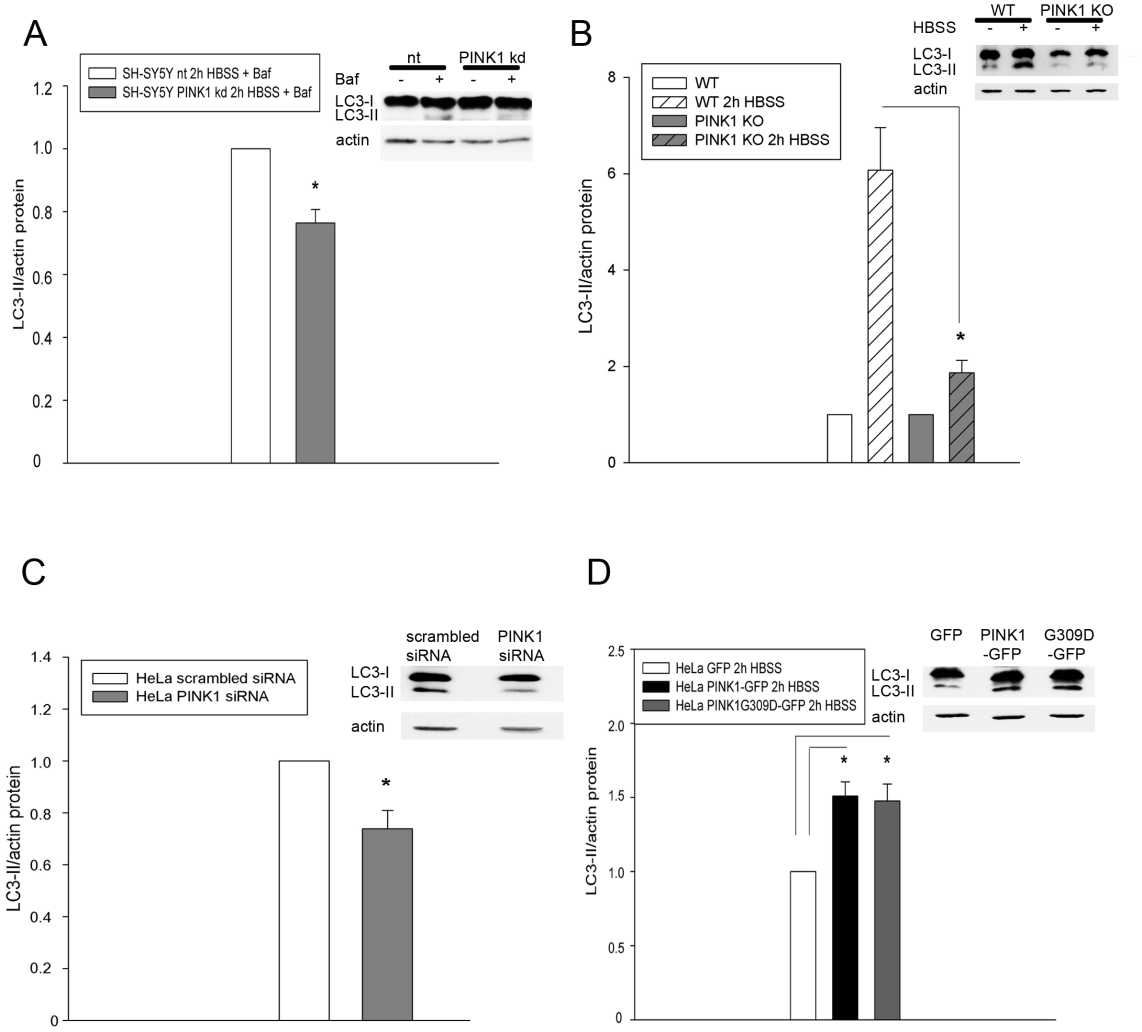
Reduced autophagy after PINK1 knockdown. 2A) SH-SY5Y cells were starved for 2 h in HBSS with Bafilomycin (+Baf) or left untreated in RPMI+5% FCS (- Baf). The LC3-II and actin content were determined by western blotting. A representative blot is shown on the right, showing only LC3-II bands in the Bafilomycin-treated samples. The LC3-II bands of Bafilomycin treated samples were normalized to actin and the relative LC3-II content of nt cells was set as 1. Cells with stable PINK1 knockdown exhibited a reduced LC3-II/actin ratio compared to the control (nt) cells; n = 4, p<0.005. 2B) Cortical neurons from 3 different isolations (10-20 DIV) of WT and PINK1 KO mice were either non-starved or starved for 2 h in HBSS and the LC3-II and actin content was determined by western blotting. A representative blot is shown on the right. The relative LC3-II content of WT and PINK1 KO mice, respectively, was set as 1. Primary neurons showed a strong upregulation of autophagy in reaction to starvation but cells derived from PINK1 KO mice exhibited a reduced LC3-II/actin ratio compared to the WT cells; n = 5, p<0.001. 2C) HeLa cells were transiently transfected with scrambled siRNA or PINK1 siRNA. After 48 h the LC3-II and actin content was determined by western blotting. A representative blot is shown on the right. The relative LC3-II content of cells transfected with scrambled siRNA was set as 1. Transient PINK1 knockdown resulted in a reduced LC3-II/actin ratio compared to the cells transfected with scrambled siRNA; n = 6; p<0.005. 2D) HeLa cells were transiently transfected with PINK1-GFP, PINK1G309D-GFP or GFP. After 24 h cells were starved for 2 h in HBSS. Afterwards the LC3-II and actin content were determined by western blotting. A representative blot is shown on the right. The relative LC3-II content of cells transfected with GFP was set as 1. Transient PINK1 or PINK1G309D overexpression resulted in an increased LC3-II/actin ratio compared to cells transfected with GFP; n = 3, PINK1: p<0.01; PINK1G309D: p<0.05.

The influence of PINK1 on LC3-II formation is controversial (see Discussion). Therefore, we investigated whether and how the observed LC3 transcript change affected LC3 protein expression. SH-SY5Y cells with or without PINK1 kd were either kept in RPMI+5% FCS or starved for 2 h in HBSS to induce autophagy and to enforce PINK1-dependent processes, and were treated with bafilomycin A to block autophagic flux. Western blotting revealed LC3-II bands only in starved cells in accordance with earlier data [16]. After starvation and bafilomycin treatment cells with PINK1 kd demonstrated significantly lower levels of LC3-II than nt cells (Fig. 2A). For validation a second cell model was employed: cortical neurons of wildtype (WT) and PINK1 knockout (KO) mice [3] from three different isolations were analyzed after different cultivation times (days in vitro) ranging from 10 to 20 days. Cortical neurons were starved for 2 h with HBSS and their LC3-II/actin ratio determined (Fig. 2B). Induction of autophagy and LC3-II formation was very strong in WT cells but significantly reduced in KO cells. In order to confirm that the observed reduction of LC3-II was directly mediated by loss of PINK1 and not a secondary, compensatory effect of stable model systems, HeLa cells were transiently transfected with a PINK1 siRNA or a scrambled siRNA. Additional stressors were not employed. After 48 h PINK1 mRNA levels were determined by RT-qPCR to verify the successful knockdown (Fig. S1 in File S1). Corroborating the data shown above, the knockdown of PINK1 in HeLa cells mediated a significant reduction of LC3-II expression (Fig. 2C).

For further evaluation, a converse experiment was designed in which PINK1-GFP, the dominant-negative mutant PINK1G309D-GFP or GFP were transiently overexpressed in HeLa cells and 24 h post transfection starved for 2 h in HBSS. Overexpression of PINK1-GFP as well as PINK1G309D-GFP resulted in significantly enhanced LC3-II levels compared to cells transfected with GFP (Fig. 2D). Taken together, these data indicate that the formation of LC3-II and thereby the induction of autophagy can be modulated by PINK1 and this becomes especially evident when cells are stressed.

### Impaired LAMP-2 expression in different cell model systems with reduced PINK1 levels

**Figure 3:**
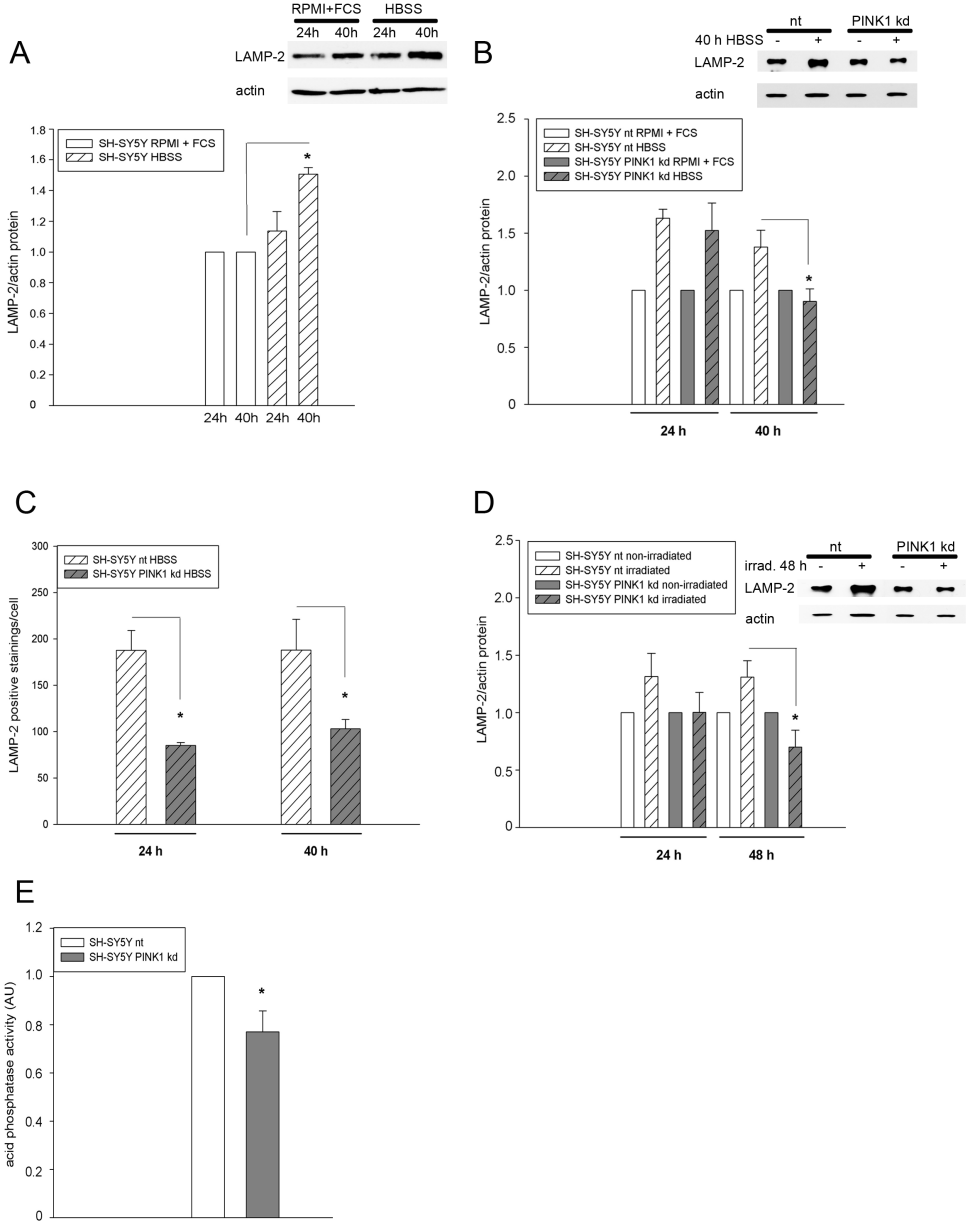
Reduced LAMP-2 expression after PINK1 knockdown. 3A) Regular, non-transduced SH-SY5Y cells were cultivated either in RPMI+10% FCS or starved in HBSS medium for 24 and 40 h. The LAMP-2 and actin content were determined by western blotting and the relative LAMP-2 content of untreated cells was set as 1. Starvation enhanced LAMP-2 protein levels; n = 3, p<0.05. 3B) Transduced nt and PINK1 knockdown (kd) SH-SY5Y cells were cultivated either in RPMI+5% FCS or starved for 24 h and 40 h in HBSS. The LAMP-2 and actin content were determined by western blotting. A representative blot after 40 h starvation in HBSS is shown on the right. The relative LAMP-2 content of untreated cells was set as 1. Cells with stable PINK1 knockdown exhibited after 40 h a reduced LAMP-2/actin ratio compared to the nt cells; n = 4, p<0.05. 3C) nt and PINK1 knockdown (kd) SH-SY5Y cells were starved for the indicated times with HBSS and afterwards stained for Lamp-2. Micrographs were taken with constant microscopical settings. LAMP-2 positive signals in each cell were quantified with ImageJ as described in Material and Methods. Knockdown of PINK1 resulted in a decreased amount of LAMP-2 positive stainings/cell after starvation; n = 2, at least 8 fields of view/condition, 89–124 cells/condition; 24 h: p<0.001; 40 h: p<0.05. 3D) nt and PINK1 knockdown (kd) SH-SY5Y cells were stained with the photo-reactive dye MTR and either irradiated for 45 min or left untreated. 24 h and 48 h after irradiation the LAMP-2 and actin content were determined by western blotting. A representative blot 48 h after irradiation is shown on the right. The relative LAMP-2 content of untreated cells was set as 1. Cells with stable PINK1 knockdown exhibited after 48 h a reduced Lamp-2/actin ratio compared to the control (nt) cells; n = 5, p<0.001. 3E) Acid phosphatase activity as parameter for lysosomal activity was quantified in nt and PINK1 knockdown (kd) SH-SY5Y cells cultivated in RPMI medium with 5% FCS. The phosphatase activity was measured and normalized to the protein content. The phosphatase activity of nt cells was set as 1. PINK1 knockdown mediated a reduction of lysosomal activity; n = 3; p<0.05.

In order to determine if the same is true for the lysosomal protein LAMP-2 the effect of starvation on the LAMP-2 expression was at first investigated in regular, non-transduced SH-SY5Y cells cultivated for 24 h and 40 h either in RPMI+10% FCS or HBSS. After 40 h a significant upregulation of LAMP-2 was observed (Fig. 3A). Next, transduced nt and PINK1 kd cells were subjected to starvation (HBSS). Already 24 h after addition of HBSS LAMP-2 protein expression was increased in nt and PINK1 kd SH-SY5Y cells (Fig. 3B), indicating that these cells react more sensitive to the loss of amino acids and growth factors than the regular, non-transduced cell line depicted in Fig. 3A. After 40 h nt cells showed the same reaction to HBSS as the regular, non-transduced SH-SY5Y cells while induction of LAMP-2 protein expression was significantly reduced in SH-SY5Y PINK1 kd cells. In contrast LAMP-1 expression was not significantly altered in these cells (Fig. S2 in File S1). Reduced LAMP-2 expression after starvation in SH-SY5Y PINK1 kd was confirmed by LAMP-2 immunocytochemistry on the single cell level (Fig. S3 in File S1 and Fig. 3C). In contrast no changes in the overall lysosomal population were visible after 40 h starvation of SH-SY5Y PINK1 kd and nt cells as shown by LysotrackerRed staining (Fig. S4 in File S2), supporting the idea of a rather specific effect of PINK1 on LAMP-2.

As second stressor targeted mitochondrial damage was employed, which initiates Parkin translocation and activates predominantly mitophagy [15]. SH-SY5Y cells with or without PINK1 kd were stained with the photoreactive dye MitotrackerRed CMX ROS (MTR) and irradiated with green light to induce oxidative stress [21]. Irradiation of MTR-stained cells resulted in mitochondrial fragmentation in a time-dependent manner followed by their degradation in lysosomes (Fig. S5 in File S3 and Fig. S6 in File S3). In accordance with earlier data [21] cells with PINK1 knockdown displayed stronger mitochondrial fragmentation than nt cells (Fig. S7 in File S3). The analysis of LAMP-2 protein levels after 45 min irradiation followed by 24 h or 48 h recovery revealed again a significantly reduced expression of LAMP-2 protein in PINK1 kd cells (Fig. 3D).

In addition, acid phosphatase activity as a parameter for lysosomal activity was determined in SH-SY5Y cells with or without PINK1 knockdown cultivated in medium with 5% FCS. SH-SY5Y cells with stable PINK1 knockdown displayed a significantly reduced acid phosphatase activity (Fig. 3E) while transient overexpression of PINK1-GFP in HeLa cells increased acid phosphatase activity (Fig. S8 in File S3). Taken together, these data identify LAMP-2 as a novel factor situated downstream of PINK1 in the response to different stressors (starvation and mitochondrial damage) and demonstrate that PINK1 influences also the lysosomal system.

### Reduced PINK1 levels influence cell metabolism and population growth

In order to determine if and how PINK1-mediated impaired autophagy influences cellular physiology, we analyzed mitochondrial morphology, energy generation and cellular growth. Stable PINK1 knockdown did not alter mitochondrial morphology of SH-SY5Y cells cultivated with 10% FCS (data not shown), 5% FCS (Fig. S5 in File S3 upper panel) or cells kept in HBSS for 2 h (data not shown) compared to nt cells in correlation with previous data (see Discussion).

**Figure 4:**
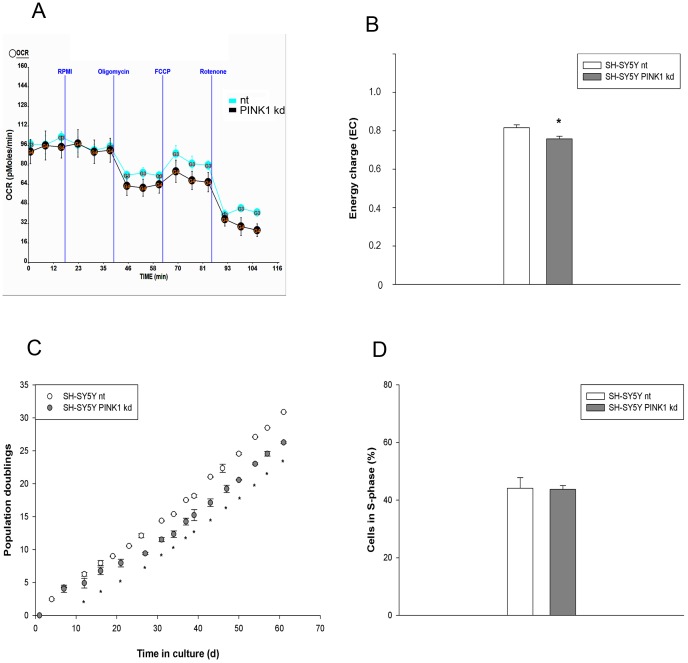
Reduced PINK1 levels influence cell metabolism and population growth. 4A) Oxygen consumption rate (OCR) was measured in nt and PINK1 knockdown (kd) SH-SY5Y cells without (nt) or with PINK1 knockdown (kd) cultivated in RPMI medium with 5% FCS. Although oxygen consumption appeared to be slightly impaired in cells with PINK1 kd, no significant changes were observed; n = 1 (quadruplicates). 4B) Energy charge (EC) of nt and PINK1 knockdown (kd) SH-SY5Y cells grown in RPMI+5% FCS was determined as detailed in Material & Methods. PINK1 knockdown resulted in a lower EC compared to nt cells; n = 5, p<0.05. 4C) nt and PINK1 knockdown (kd) SH-SY5Y cells were grown for 2 months in RPMI+5% FCS and their population doublings were determined. Starting from day 12 cells with PINK1 kd exhibited impaired cell population growth; n = 1 (triplicates); * p<0.05 (day 12) - p<1×10^−6^ (day 61). 4D) SH-SY5Y cells were grown in RPMI+5% FCS and the amount of nt and PINK1 knockdown (kd) cells in S-phase was determined by BrdU incorporation. No differences in cell proliferation were visible between cells without (nt) and with PINK1 kd; n = 3.

As functional physiological parameter oxygen consumption was determined in adherent SH-SY5Y cells with light trophic stress (RPMI with 5% FCS). Although respiration appeared to be slightly impaired in cells with PINK1 knockdown, no significant alteration could be observed due to the variability of the measurements (Fig. 4A). Therefore, in addition the AMP, ADP and ATP content of nt and PINK1 kd cells grown in medium with 5% FCS was determined and their energy charge (EC) calculated. SH-SY5Y cells with reduced PINK1 content exhibited a slight but significant decrease of the EC (Fig. 4B) under mild trophic stress.

Cell proliferation was investigated as parameter for energy availability. Growth curves of SH-SY5Y cells cultivated with 5% FCS show that cell population growth of PINK1 kd cells was impaired compared to control nt cells (Fig. 4C). Cell cycle progression was determined by quantifying the percentage of cells in S-phase after BrdU incorporation. The amount of cells in S-phase was not influenced by the PINK1 content (Fig. 4D), indicating that the cell cycle itself was not modulated by PINK1.

### Impaired cell population growth of PINK1 kd cells is due to increased apoptosis

**Figure 5:**
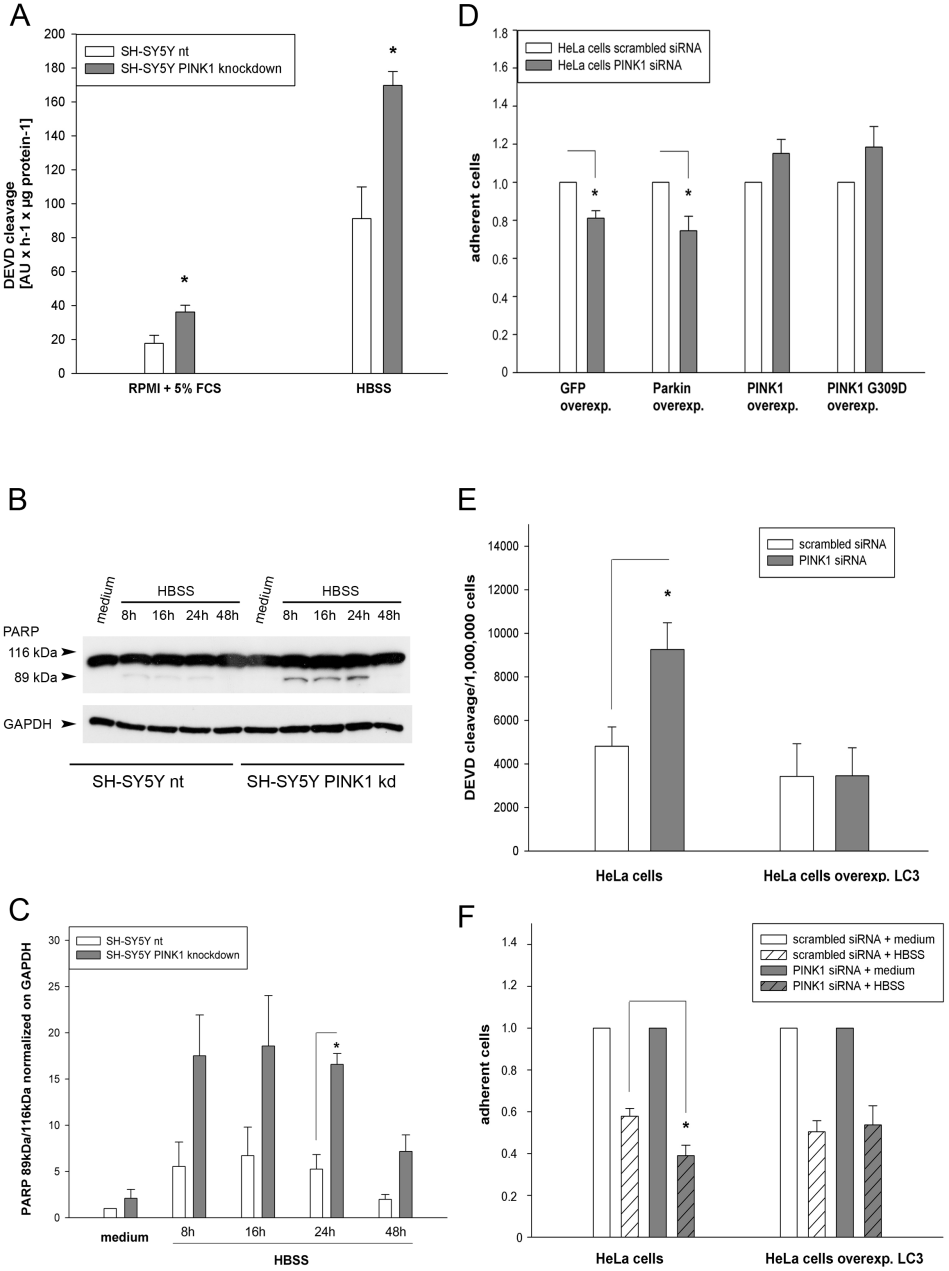
Increased apoptosis in cells with PINK1 knockdown. 5A) nt and PINK1 knockdown (kd) SH-SY5Y cells were either kept in RPMI medium with 5% FCS or starved with HBSS for 12 h. DEVD cleavage as parameter for caspase-3 activity was analyzed and normalized to the protein content. Cells with PINK1 knockdown demonstrated a significantly increased caspase-3 activity under both conditions; n = 4; RPMI+5% FCS: p<0.05; HBSS: p<0.01. 5B) nt and PINK1 knockdown (kd) SH-SY5Y cells were either kept in RPMI medium with 5% FCS for 48 h or starved with HBSS for the indicated time points. In the representative western blot the appearance of the PARP 89 kDa cleaved product is depicted as well as GAPDH for normalizing. 5C) nt and PINK1 knockdown (kd) SH-SY5Y cells were either kept in RPMI medium with 5% FCS for 48 h or starved with HBSS for the indicated time points. For quantification the cleaved PARP 89 kDa product was normalized to the full length 113 kDa PARP and the resulting value normalized to GAPDH. Cells with PINK1 knockdown demonstrated increased PARP cleavage that was significant after 24 h starvation; n = 4; 24 h: p<0.005. 5D) HeLa cells were transfected with scrambled siRNA or PINK1 siRNA and GFP or GFP-Parkin or PINK1-GFP or PINK1G309D-GFP. After transfection cells were starved for additional 24 h with HBSS and afterwards the amount of adherent cells was determined. The number of adherent cells transfected with scrambled siRNA and the indicated plasmid was set as 1. Starvation-mediated increased cell loss after PINK1 knockdown could be rescued by PINK1 or PINK1G309D-GFP but not by GFP or Parkin; GFP: n = 5, p<0.005; Parkin: n = 3, p<0.05; PINK1: n = 5, ns; PINK1G309D-GFP: n = 3, ns. 5E) HeLa cells and HeLa cells stably overexpressing LC3 were transfected with scrambled siRNA or PINK1 siRNA. After 48 h cells were starved in HBSS for additional 24 h and afterwards DEVD cleavage as parameter for caspase-3 activity was analyzed and normalized to 1,000,000 cells. PINK1 knockdown increased caspase-3 activity significantly, which was prevented by LC3 overexpression; n = 3; p<0.05. 5F) HeLa cells and HeLa cells stably expressing LC3 were transfected with scrambled or PINK1 siRNA. 48 h post transfection cells were either left untreated (+ medium) or starved (+HBSS) for additional 24 h and afterwards the amount of adherent cells was determined. The amount of non-starved cells was set as 1. PINK1 knockdown resulted in elevated cell loss, which was prevented by LC3 overexpression; HeLa: n = 6, p<0.05; HeLa LC3: n = 5.

Elevated apoptotic rates would explain the reduced growth of the SH-SY5Y PINK1 kd cell population despite their unaltered cell cycle progression. Therefore, DEVD cleavage as parameter for caspase-3 activity was analyzed in cells with or without PINK1 kd. SH-SY5Y cells with PINK1 kd exhibited enhanced caspase-3 activity in medium with 5% FCS compared to nt cells (Fig. 5A). When trophic stress was increased by starving cells 12 h in HBSS, caspase-3 activity of nt and PINK1 kd cells was accordingly elevated, but this increase was much stronger in PINK1 kd cells. In accordance with these data an increased appearance of the cleaved PARP product (89 kDa) could be detected by western blotting in nt and PINK1 kd cells (Fig. 5B) but PINK1 kd cells contained a significantly higher amount of the cleaved 89 kDa PARP product in the later stages of starvation (Fig. 5C).

Decreased autophagy is known to result in increased apoptosis [22]–[25], thus we hypothesized that the PINK1-mediated impairment of the autophago-lysosomal pathway had caused or at least contributed to the elevated apoptotic rates. Therefore, we set up different rescue experiments to confirm direct links between PINK1, the autophago-lysosomal pathway and apoptosis. HeLa cells were transiently double transfected with a PINK1 siRNA or a control scrambled siRNA and GFP or PINK1-GFP or PINK1G309D-GFP or GFP-Parkin. After transfection cells were starved for 24 h and the number of remaining, adherent cells was quantified. Transient knockdown of PINK1 in GFP transfected HeLa cells resulted in an increased cell death after starvation that could by rescued by overexpression of PINK1 or PINK1G309D but not Parkin (Fig. 5D).

Finally, HeLa cells and stably LC3 overexpressing HeLa cells were transfected with a PINK1 siRNA or scrambled siRNA. 48 h after transfection cells were subjected to 24 h starvation in HBSS. The reduced expression of PINK1 in HeLa cells mediated an elevated activity of caspase-3 (Fig. 5E left side) in accordance to the data obtained with SH-SY5Y cells (Fig. 5A). This increased apoptosis rate resulted again also in a significant reduction of adherent cells (Fig. 5F left side). LC3 overexpressing HeLa cells were protected against cell death after PINK1 knockdown and starvation as demonstrated by their lower caspase-3 activity (Fig. 5E right side) and the higher number of surviving cells (Fig. 5F right side). In contrast starvation-induced cell death after PINK1 knockdown could not be rescued in HeLa cells stably overexpressing LAMP-1 (Fig. S9 in File S3). Taken together, these data confirm that PINK1-mediated impairment of specific autophago-lysosomal factors results in apoptosis under stress.

## Discussion

In the recent years a role for PINK1 upstream of Parkin in mitophagy has emerged. Loss of functional PINK1 or Parkin impairs mitophagy and results in mitochondrial dysfunction [12]–[15]. This could lead to increased cellular vulnerability and may contribute to the development and progression of PD. Recent results show that expression of PINK1 and Parkin is induced by trophic stress [16]–[17]. According to these data it seems possible that PINK1, in addition to mitophagy, has also a functional role in stress-induced autophagy.

In order to evaluate this hypothesis we generated a stable PINK1 knockdown in SH-SY5Y neuroblastoma cells, an established cell model system for PD. A careful analysis of transcript levels of autophagy-related genes revealed that the loss of PINK1 resulted only under slight trophic stress (medium with 5% FCS) in a significant downregulation of key genes of the autophago-lysosomal pathway including Beclin and LC3. Recent data showed already that PINK1 interacts with Beclin [19] and LC3 [20] on the protein level and now we can expand this interaction to the genetic level. Accordingly, the loss of PINK1 together with an additional stressor resulted in a reduced amount of LC3-II in stable and transient cell model systems, while overexpression of PINK1 enhanced LC3-II levels. These data are in agreement with results presented by [19] who showed that PINK1 levels correlate positively with the LC3-II content and with [26] who observed a reduction of LC3-positive vacuoles in stressed cells after PINK1 knockdown. In contrast, [27] and [28] observed an increased amount of LC3-II after knockdown of PINK1. Based on our data it seems possible that these conflicting results can be at least partly related to different stress levels during transfection and culturing. In accordance with [19] our overexpression of the PINK1G309D mutant increased the LC3-II/actin ratio similar to the PINK1 overexpression.

Similar to LC3-II, the effect of PINK1 knockdown on LAMP-2 mRNA and protein only became apparent in stressed cells: LAMP-2 but not LAMP-1 levels were significantly reduced after HBSS treatment or mitochondrial-targeted oxidative stress in cells with PINK1 knockdown. LAMP-2 protein expression is downregulated in brain tissue [29] and also in peripheral leukocytes [30] of PD patients, which supports a role of LAMP-2 in the pathogenesis of PD.

The effects of impaired autophagy in PINK1 knockdown/knockout cells were investigated in regard to mitochondrial biology. Mitochondrial morphology was not altered by PINK1 knockdown in untreated SH-SY5Y cells or cells under trophic stress, correlating with earlier works that observed in human cells just little or no effect of PINK1 downregulation [3], [19], [21], [31], [32] or upregulation [33] on mitochondrial morphology. In contrast other authors found increased mitochondrial shortening respective elongation after loss of functional PINK1 in human cells [27], [34]–[38]. The reasons behind this ongoing discrepancy are still unclear but they could at least partially stem from differences of cell model systems and stress levels. Despite the unchanged morphology energy production of SH-SY5Y cells with PINK1 knockdown was reduced by 8% corroborating earlier reports showing that the loss of PINK1 impairs energy generation [3], [32], [39].

Decreased autophagy correlates with an increase in apoptosis [22]–[25] and knockout of the autophagy genes ATG5 or ATG7 in murine neurons results in neuronal cell death [40]–[42]. The link between autophagy and apoptosis is supported by our data as the loss of PINK1 in two different cell models leads to increased apoptosis that could be rescued by LC3 overexpression. The elevated apoptotic rates of cells with PINK1 knockdown correlated well with the observed impaired cell population growth, especially since no effect on cell cycle progression (amount of cells in S-phase) could be detected. Also astrocytes of PINK1 knockout mice showed impaired population growth [39], supporting the importance of PINK1 also for proliferating cells. The apoptosis-promoting effect of PINK1 knockdown is in agreement with earlier data showing a positive correlation between PINK1 levels and apoptotic rates in different model systems [26], [43]–[47]. Against our expectations, Parkin-GFP was unable to reduce apoptotic rates after loss of PINK1, indicating that the PINK1-mediated effects described here may occur in a Parkin-independent manner. In contrast, overexpression of PINK1 or the PINK1 mutant PINK1G309D was able to rescue PINK1 knockdown-mediated cell death during starvation. These data are in accordance with our findings that overexpression of PINK1G309D did not impair LC3-II formation during starvation and indicate that the PINK1G309D mutation has no effect on starvation-induced PINK1-mediated autophagy induction or cell death after prolonged starvation.

Taken together, we could show that PINK1 modulates the autophago-lysosomal pathway under stress and that PINK1-mediated reduction of autophagic key factors results in increased cell death. This represents an additional and apparently Parkin-independent pathway, in which the loss of PINK1 could contribute to the development and progression of PD.

## Material and Methods

### Cultivation of cells

SH-SY5Y cells (European Collection of Cell Cultures; Sigma-Aldrich) were cultivated in RPMI 1640 medium containing 2 g/l glucose and 5% or 10% FCS. HeLa cells (Deutsche Sammlung von Mikroorganismen und Zellkulturen GmbH) were cultivated in Minimal Essential Medium with Earle's salts containing 10% FCS and 1% non essential amino acids. Primary cortical neurons were isolated from 1–4 d old mice as described previously [3] and incubated with Neurobasal medium for 10–20 days before performing experiments. All cells were kept at 37°C with 5% CO_2_ and 95% air.

### Generation of SH-SY5Y cells with stable PINK1 knockdown

SH-SY5Y cells were transduced using Mission shRNA lentiviral particles (Sigma) with a MOI of 15. The lentivirus coded either for a non-targeted (nt) shRNA (SHC002V) or an shRNA directed against PINK1 (TRCN0000007101). For selection of successfully transduced cells Puromycin (final concentration 1 µg/ml) was used. After 14 d the PINK1 mRNA expression of transduced cells was 50% reduced in comparison to the nt cells. Therefore, PINK1 knockdown cells were subjected to one round of subcloning. This procedure resulted in two clones with stronger reduction of PINK1 expression but only one of them demonstrated a stable downregulation and was therefore used for the experiments.

### Starvation and irradiation of cells

Cells were kept for the indicated times in HBSS medium with 1 g/l glucose but without serum or amino acids. Irradiation of cells was described before [21]. Briefly, cells were stained with MitoTrackerRed CMX ROS (final concentration 25 nM) for 1 h and irradiated for the indicated times with green light.

### Constructs and transfection

Cloning of PINK1-GFP [21], PINK1G309D-GFP [21], GFP-Parkin [15], GFP-LC3 [15], and LAMP-1-GFP [15] was described before. Transient knockdown of PINK1 was achieved with PINK1 antisense RNA (HS_PINK1_4_HP_Validated siRNA, Qiagen) and Allstars Negative Control (scrambled) siRNA (Qiagen) was used as control. Transient transfection of SH-SY5Y cells was achieved by electroporation with the Cell Line V transfection kit (Amaxa). HeLa were transiently transfected by using Effectene (Qiagen). Stable transfected GFP-LC3 and LAMP-1-GFP expressing HeLa cells were generated by selection with G418.

### RNA isolation and quantitative RT-PCR

Rapamycin (Sigma) was used in a final concentration of 0.5 µM and LY294002 (Jena Bioscience) in a final concentration of 25 µM. RNA was isolated with the RNeasy mini kit (Qiagen) and then treated with DNase I. cDNA was synthesized with SuperScript III reverse transcriptase using oligo(dT)_20_ and random primers (Invitrogen). cDNA from 30 ng RNA were utilized in a 20 µl reaction volume using the StepOnePlus Real-Time PCR System and the appropriate TaqMan gene expression assays (Applied Biosystems): PINK1 (Hs00260868_m1), ATG5 (Hs00355494_m1), ATG6 (Hs00186838_m1), ATG7 (Hs00197348_m1), ATG8E (Hs01076567_g1), p62/SQSTM1 (Hs00177654_m1), PARK2 (Hs01038318_m1) LAMP-1 (Hs00931464_m1), LAMP-2 (Hs00903587_m1), Mfn1 (Hs00566851_m1S), Mfn2 (Hs00208382_m1), Opa1 (Hs01047019_m1), Drp1 (Hs00247147_m1), Fis1 (Hs00211420_m1) March5 (Hs00215155_m1), ATP13A2 (Hs00223032_m1) SOD2 (Hs00167309_m1), HIF1A (Hs00936368_m1) NFKBIA (Hs00153283_m1), FOXO3A (Hs00921424_m1), SGK1 (Hs00178612_m1), ACTB (Hs99999903_m1), GAPDH (Hs99999905_m1). mRNA expression was normalized to the TATA box binding protein (TBP: Hs99999910_m1). Relative expression changes were calculated with the 2^-ΔΔCt^ method [48].

### Western blotting

Bafilomycin A (Sigma) was used in a final concentration of 10 nM in HBSS medium for 2 h. Lysates of proteins were separated by SDS-PAGE and transferred by wet blotting onto a nitrocellulose membrane. PINK1 (Novus Biological), LC3 (Sigma) and PARP (Cell Signaling) were detected with polyclonal antibodies and LAMP-1 (BD Transduction Laboratories), LAMP-2 (BD Transduction Laboratories) and beta-actin (Sigma) were detected with monoclonal antibodies. The SuperSignal West Pico Chemiluminescent Substrate (Thermo) was used for detection of horseradish peroxidase activity on the secondary antibodies and bands were quantified with the program ImageJ.

### Microscopy

Mitochondria were stained with MitoTracker Red CMX ROS (MTR) (final concentration 25 nM) for 1 h and afterwards fixed with 4% paraformaldehyde. Cells were incubated with an anti-LAMP-2 antibody (BD Bioscience Pharmingen) and analyzed with a Leica TCS SP5 confocal laser scanning microscope (CLSM) with the objective HCX PL APO lambda blue 63.0x, 1.40 OIL UV that was controlled by the LAS AF scan software (version 1.8.2) (Leica). CLSM pictures were processed with IMARIS 6.0.0 (BITPLANE Scientific solutions). Counting of LAMP-2 positive structures on the single cell level was performed with ImageJ using a constant threshold (Analyze Particles: pixel size 1–300, circularity 0.00–1.00). For visualization of lysosomes cells were incubated with LysotrackerRed (final concentration 75 nM) for 1 h and live imaging was performed with an Axiovert 200 M fluorescence microscope (Zeiss) in a chamber warmed to 37°C.

### Cell proliferation

Population doublings in growth curves were determined by the following equation: Population doubling  = 3.32 * (log10 UCY - log10 I) +X (where UCY is the number of cells at the end of the passage; I the number of cells that were seeded at the beginning of the passage and X the previous population doubling number. To determine the number of cells in S-phase 10 µM 5-Bromo-2-deoxy-uridine (BrdU; Invitrogen) was added to the cells for 40 min followed by fixation with 70% ethanol. Cells were then treated with 2 M HCl, washed with 0.5% Tween 20 and incubated with an anti-BrdU antibody (Invitrogen). As secondary antibody an anti-mouse FITC-conjugated antibody (Dianova) was used. Finally nuclei were stained with Hoechst 33258 to visualize all nuclei.

### Lysosomal activity

The activity of acid phosphatase, a key enzyme of the lysosomes, was used as a parameter for lysosomal activity. All experiments were performed in triplicates in a 96 well plate. 300,000 cells were lysed in lysis buffer [49] and added to the substrate provided with the Acid Phosphatase Assay Kit (Sigma). After 30 min incubation at 37°C the absorbance of p-Nitrophenol was determined at 405 nm in an ELISA reader (Tecan) and normalized to the cell number (overexpressing HeLa cells) or to the total protein content (SH-SY5Y cells).

### Respirometry

Mitochondrial oxygen consumption was measured by respirometry using the XF24 Analyzer (Seahorse Bioscience). 60,000 SH-SY5Y cells were seeded into an XF24 plate and incubated over night at 37°C and 5% CO_2_. Cells were kept for the experiment in RPMI without FCS and respiration was measured under four conditions: RPMI alone, RPMI with oligomycin (final concentration 1 µM), RPMI with CCCP (final concentration 3 µM) and RPMI with rotenone (final concentration 2 µM) according to the manufacturer's instructions.

### Energy charge

For the determination of the cellular energy charge (EC), adenine nucleotides were extracted from cells using a freeze-thaw procedure adapted from Spielmann et al. [50]. To attenuate the activity of various cellular ATP hydrolyzing enzymes, cells were treated for 15 s with microwave irradiation at 560 W, and a cocktail of inhibitors against ATPases (bafilomycin, oligomycin and ouabain (final concentrations 10 nM) was added prior to the freeze-thaw procedure. ATP was quantitatively determined by bioluminescence using firefly luciferase and its substrate D-luciferin. With this assay, ADP and AMP were also quantified after conversion into ATP by pyruvate kinase and pyruvate kinase combined with myokinase, respectively. ATP concentrations were directly calculated from the respective samples, while the concentrations of ADP and AMP were obtained as C_ADP_ = C_(ATP+ADP)_- C_ATP_ and C_AMP_ = C_(ATP+ADP+AMP)_ - C_(ATP+ADP)_. EC = C_ATP_+(0.5* C_ADP_)/C_(ATP+ADP+AMP)_.

### Caspase-3 activity

Assessment of caspase-3 activity has been described before [11]. All experiments were performed in quadruplicates in a 96 well plate. Cells were lysed and the cell lysate was mixed with reaction buffer containing the caspase-3 substrate Ac-DEVD-AMC. Fluorescence intensity was measured in a fluorescent plate reader (Tecan) (excitation 380 nm, emission 465 nm) and normalized to whole protein content.

### Statistics

Results are expressed as means ±SEM of n experiments, apart from the growth curve where SD was used. ANOVA was used to compare sets of data. Differences were considered statistically significant when p<0.05.

## Supporting Information

File S1 **Figure S1,** *Transient PINK1 knockdown in HeLa cells.* HeLa cells were transiently transfected with scrambled siRNA or PINK1 siRNA. Afterwards PINK1 mRNA content was determined by RT-qPCR. Transient PINK1 knockdown resulted in a reduced PINK1 mRNA expression compared to the control; n = 5; p<0.001. **Figure S2**, *LAMP-1 expression is not altered by starvation*. nt and PINK1 knockdown (kd) SH-SY5Y cells were cultivated either in RPMI+5% FCS or starved for 40 h in HBSS. The LAMP-1 and actin content were determined by western blotting. The relative LAMP-1 content of untreated cells was set as 1. PINK1 knockdown had no effect on LAMP-1 expression after starvation; n = 3. **Figure S3**, *PINK1 knockdown results in reduced LAMP-2 staining after starvation*. nt and PINK1 knockdown (kd) SH-SY5Y cells were starved for the indicated times with HBSS and afterwards stained for Lamp-2. Micrographs were taken with constant microscopical settings. Cells with stable PINK1 knockdown showed a reduced LAMP-2 reactivity compared to control (nt) cells; bar  = 10 µm.
(TIF)

File S2 **Figure S4,** *PINK1 knockdown does not affect lysosomal population.* SH-SY5Y cells nt (two representative pictures on the left) and PINK1 knockdown (kd) (two representative pictures on the right) were starved for 40 h in HBSS. Lysosomes were visualized by staining with LysotrackerRed. No difference between cells without and with PINK1 knockdown were visible; n = 3, bar  = 10 µm.
(TIF)

File S3 **Figure S5,** *Mitochondrial fragmentation after irradiation*. nt and PINK1 knockdown (kd) SH-SY5Y cells cultivated in RPMI medium with 5% FCS were stained with MTR and then either irradiated or non-irradiated for 45 min. After 8 h mitochondrial morphology was depicted by CLSM. Irradiation resulted in mitochondrial fragmentation but no changes induced by PINK1 knockdown are apparent; bar  = 7 µm. **Figure S6**, *Degradation of fragmented mitochondria*. In order to demonstrate lysosomal degradation of fragmented mitochondria SH-SY5Y nt cells stained with MTR (panel on the left) were co-stained for LAMP-2 (panel in the middle). The merged image (panel on the right) shows clear co-localization of the MTR and the LAMP-2 staining (arrowheads); bar  = 3 µm. **Figure S7**, *Establishment of an irradiation regime for SH-SY5Y cells*. SH-SY5Y cells were stained with the photo-reactive dye MTR and irradiated for 30 or 45 min. 8 h after irradiation the mitochondrial morphology was determined by microscopy. 45 min irradiation resulted in mitochondrial fragmentation in about half of the cell population; n = 1, at least 100 cells/condition in at least 10 fields of view. **Figure S8**, *PINK1 overexpression increases acid phosphatase activity*. HeLa cells were transiently transfected with PINK1-GFP or GFP and the acid phosphatase activity as parameter for lysosomal activity of 200.000 cells was measured. The phosphatase activity of GFP transfected cells was set as 1. Transient PINK1 overexpression mediated an elevation of lysosomal activity; n = 4; p<0.005. **Figure S9**, *LAMP-1 overexpression does not protect against starvation-induced cell death after PINK1 knockdown*. HeLa cells stably expressing LAMP-1 were transfected with scrambled siRNA or PINK1 siRNA. 48 h post transfection cells were either left untreated (medium) or starved (+HBSS) for additional 24 h. Afterwards the amount of adherent cells was determined. The amount of non-starved cells was set as 1. PINK1 knockdown resulted in elevated cell loss compared to cells transfected with scrambled siRNA; n = 4, p<0.05.
(TIF)

File S4 **Table S1,** *No effects of PINK1 on non-lysosomal genes in cells cultivated with 5% FCS.* Different genes not involved in mitochondrial dynamics or the autophago-lysosomal pathway and two house-keeping genes were analyzed by RT-qPCR in control (nt) and PINK1 knockdown SH-SY5Y cells cultivated with 10% or 5% FCS. The relative gene expression in nt cells was set in both conditions as 1. No significant changes appeared in cells kept in 5%.
(DOC)
