# Supplementary figures and images for: Loss of PINK1 Impairs Stress-Induced Autophagy and Cell Survival
Source: PLoS One. 2014 Apr 21;9(4):e95288. doi: 10.1371/journal.pone.0095288 (PMC3994056; doi:10.1371/journal.pone.0095288)

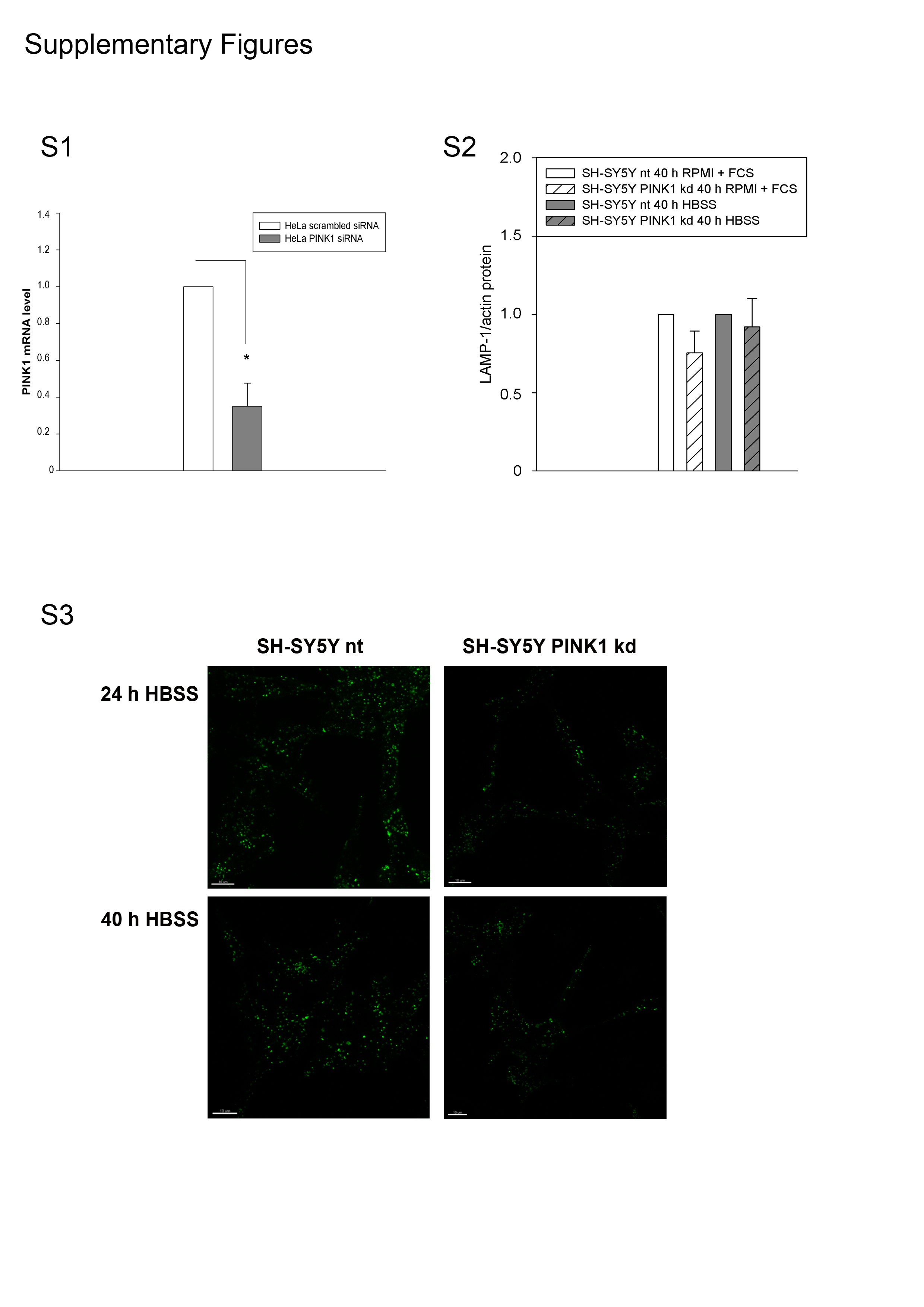

Supplement: File S1 — Figure S1, Transient PINK1 knockdown in HeLa cells. HeLa cells were transiently transfected with scrambled siRNA or PINK1 siRNA. Afterwards PINK1 mRNA content was determined by RT-qPCR. Transient PINK1 knockdown resulted in a reduced PINK1 mRNA expression compared to the control; n = 5; p<0.001. Figure S2, LAMP-1 expression is not altered by starvation. nt and PINK1 knockdown (kd) SH-SY5Y cells were cultivated either in RPMI+5% FCS or starved for 40 h in HBSS. The LAMP-1 and actin content were determined by western blotting. The relative LAMP-1 content of untreated cells was set as 1. PINK1 knockdown had no effect on LAMP-1 expression after starvation; n = 3. Figure S3, PINK1 knockdown results in reduced LAMP-2 staining after starvation. nt and PINK1 knockdown (kd) SH-SY5Y cells were starved for the indicated times with HBSS and afterwards stained for Lamp-2. Micrographs were taken with constant microscopical settings. Cells with stable PINK1 knockdown showed a reduced LAMP-2 reactivity compared to control (nt) cells; bar = 10 µm. (TIF) [file pone.0095288.s001.tif]

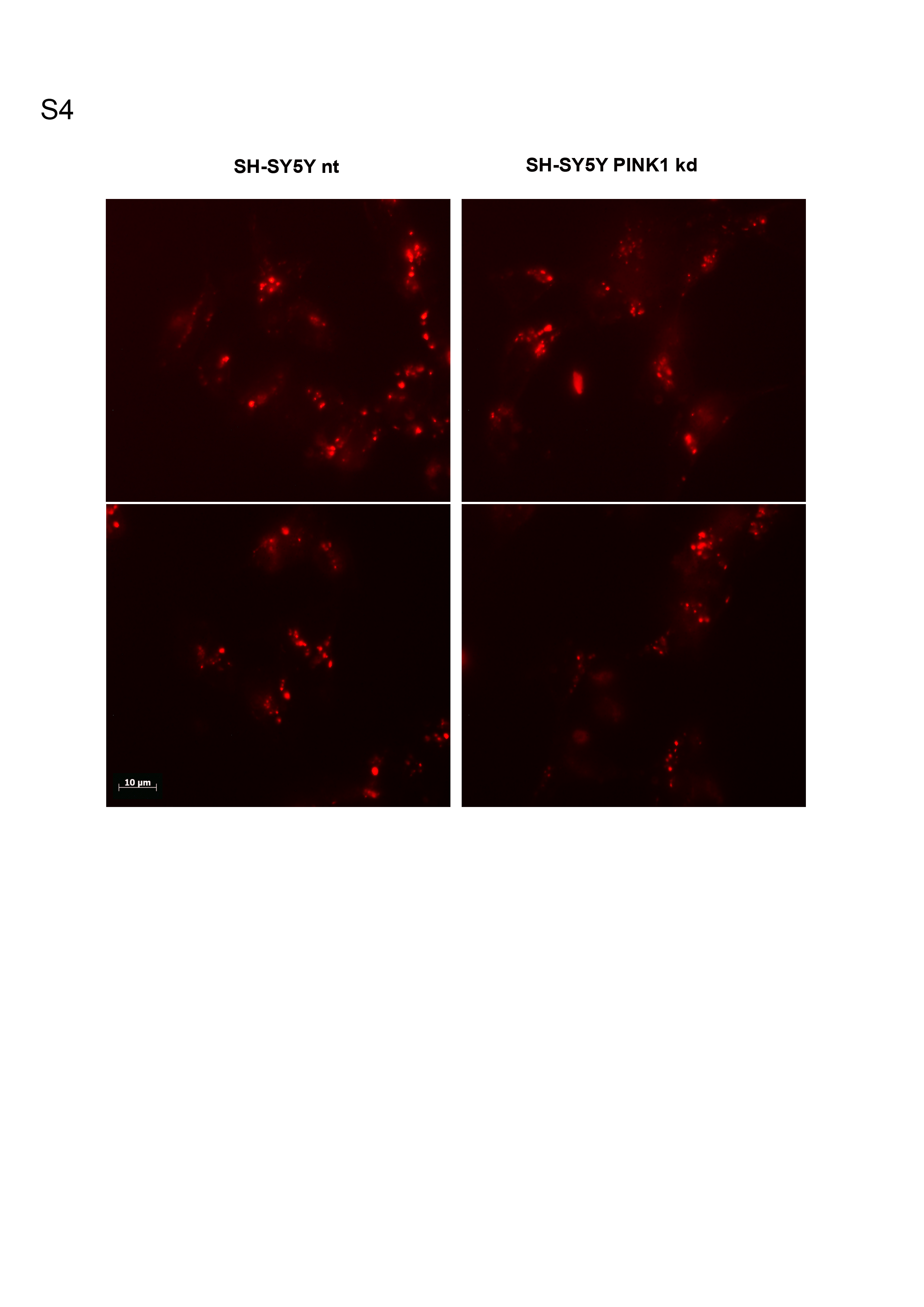

Supplement: File S2 — Figure S4, PINK1 knockdown does not affect lysosomal population. SH-SY5Y cells nt (two representative pictures on the left) and PINK1 knockdown (kd) (two representative pictures on the right) were starved for 40 h in HBSS. Lysosomes were visualized by staining with LysotrackerRed. No difference between cells without and with PINK1 knockdown were visible; n = 3, bar = 10 µm. (TIF) [file pone.0095288.s002.tif]

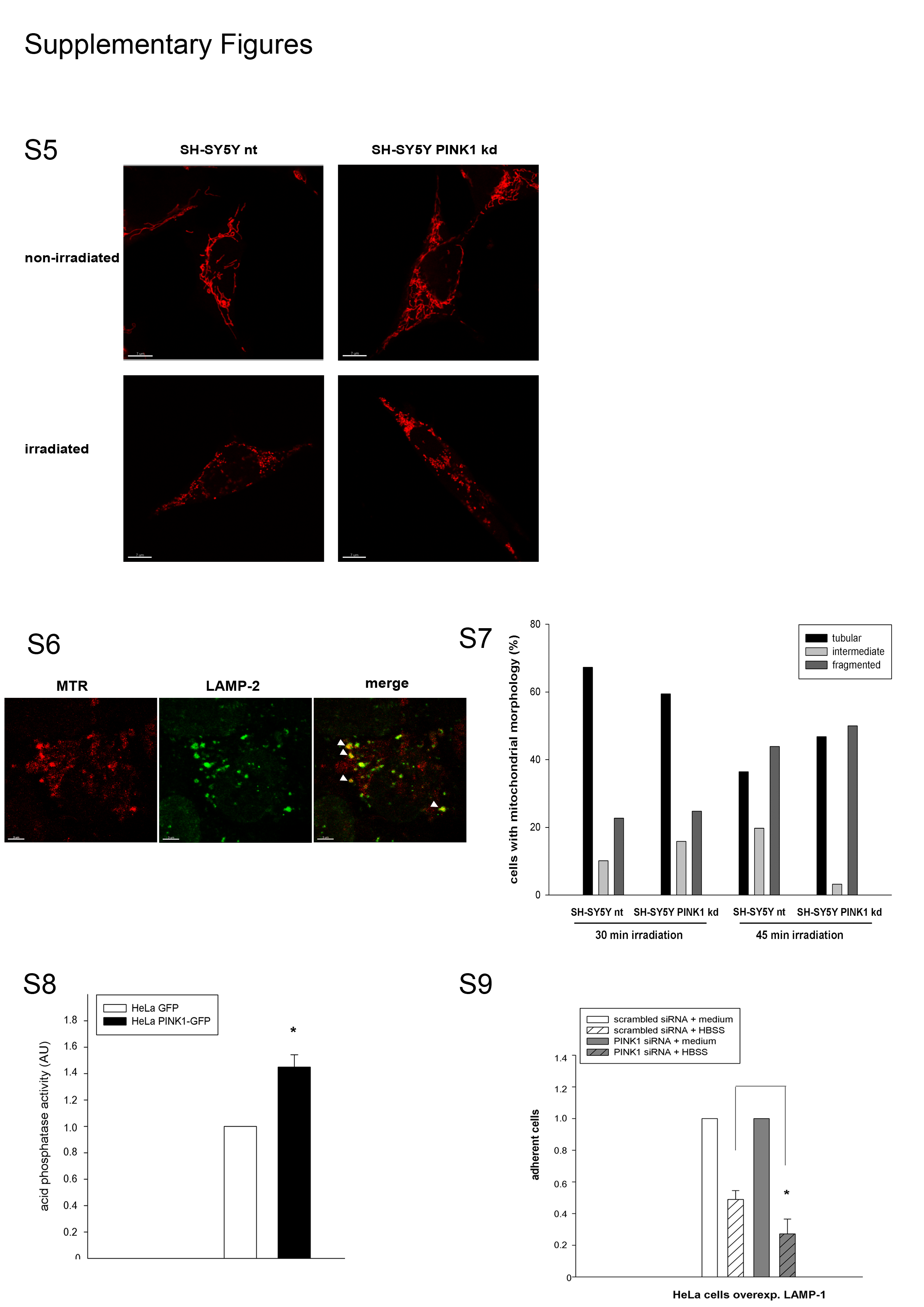

Supplement: File S3 — Figure S5, Mitochondrial fragmentation after irradiation. nt and PINK1 knockdown (kd) SH-SY5Y cells cultivated in RPMI medium with 5% FCS were stained with MTR and then either irradiated or non-irradiated for 45 min. After 8 h mitochondrial morphology was depicted by CLSM. Irradiation resulted in mitochondrial fragmentation but no changes induced by PINK1 knockdown are apparent; bar = 7 µm. Figure S6, Degradation of fragmented mitochondria. In order to demonstrate lysosomal degradation of fragmented mitochondria SH-SY5Y nt cells stained with MTR (panel on the left) were co-stained for LAMP-2 (panel in the middle). The merged image (panel on the right) shows clear co-localization of the MTR and the LAMP-2 staining (arrowheads); bar = 3 µm. Figure S7, Establishment of an irradiation regime for SH-SY5Y cells. SH-SY5Y cells were stained with the photo-reactive dye MTR and irradiated for 30 or 45 min. 8 h after irradiation the mitochondrial morphology was determined by microscopy. 45 min irradiation resulted in mitochondrial fragmentation in about half of the cell population; n = 1, at least 100 cells/condition in at least 10 fields of view. Figure S8, PINK1 overexpression increases acid phosphatase activity. HeLa cells were transiently transfected with PINK1-GFP or GFP and the acid phosphatase activity as parameter for lysosomal activity of 200.000 cells was measured. The phosphatase activity of GFP transfected cells was set as 1. Transient PINK1 overexpression mediated an elevation of lysosomal activity; n = 4; p<0.005. Figure S9, LAMP-1 overexpression does not protect against starvation-induced cell death after PINK1 knockdown. HeLa cells stably expressing LAMP-1 were transfected with scrambled siRNA or PINK1 siRNA. 48 h post transfection cells were either left untreated (medium) or starved (+HBSS) for additional 24 h. Afterwards the amount of adherent cells was determined. The amount of non-starved cells was set as 1. PINK1 knockdown resulted in elevated cell loss co [file pone.0095288.s003.tif]
