# Supplementary material for: Loss of PINK1 Impairs Stress-Induced Autophagy and Cell Survival
Source: PLoS One. 2014 Apr 21;9(4):e95288. doi: 10.1371/journal.pone.0095288 (PMC3994056; doi:10.1371/journal.pone.0095288)
Supplement: File S4 — Table S1, No effects of PINK1 on non-lysosomal genes in cells cultivated with 5% FCS. Different genes not involved in mitochondrial dynamics or the autophago-lysosomal pathway and two house-keeping genes were analyzed by RT-qPCR in control (nt) and PINK1 knockdown SH-SY5Y cells cultivated with 10% or 5% FCS. The relative gene expression in nt cells was set in both conditions as 1. No significant changes appeared in cells kept in 5%. (DOC) [file pone.0095288.s004.doc]

| **Gene** | **PINK1 kd cells in 10% FCS**  Fold-change *p*-value | **PINK1 kd cells in 5% FCS**  Fold-change *p*-value |
| --- | --- | --- |
| ATP13A2 | 1.335 ns | 0.726 ns |
| SOD2 | 1.114 ns | 0.664 ns |
| HIF1A | 1.143 0.0062 | 0.817 ns |
| INFKBIA | 0.795 ns | 0.461 ns |
| FOXO3A | 1.244 ns | 0.891 ns |
| SGK1 | 1.004 ns | 0.693 ns |
| ACTB | 1.124 ns | 1.219 ns |
| GAPDH | 0.992 ns | 1.053 ns |
